# Compensatory role of Neuroglobin in nervous and non-nervous cancer cells in response to the nutrient deprivation

**DOI:** 10.1371/journal.pone.0189179

**Published:** 2017-12-07

**Authors:** Marco Fiocchetti, Manuela Cipolletti, Maria Marino

**Affiliations:** Department of Science, University of Roma Tre, Viale Guglielmo Marconi, Roma, Italy; University of Naples 2, ITALY

## Abstract

Environmental factors or adverse growth conditions that may reduce cell function or viability are considered stress. The cell ability to sense and respond to environmental stresses determine its function and survival destiny. We recently defined Neuroglobin (NGB), a heme-protein, as a compensatory protein in the 17β-Estradiol (E2) anti-apoptotic activity and as a sensor of oxidative stress in both neurons and breast cancer cells. Here, the possibility that NGB levels could represent a pivotal regulator of integrated response of cancer cells to stress has been evaluated. Data obtained in neuroblastoma and in breast cancer cell lines evidence that nutrient deprivation significantly up-regulated NGB levels at different time points. However, the analysis of autophagy activation led to exclude any possible role of stress- or E2-induced NGB in the upstream regulation of general autophagy. However, the over-expression of Flag-NGB in ERα stable transfected HEK-293 cells completely affects nutrient deprivation-induced decrease in cell number. In addition, reported results indicate that modulation of the anti-apoptotic Bcl-2 level may play a key role in the protective NGB function against energetic stress. Overall, these data define a role of NGB as compensatory protein in the cell machinery activated in response to stress and as general stress adaptation marker of cancer cells susceptible to oxidative stress, oxygen and, as demonstrated here for the first time, even to nutrient willingness. Despite the lacking of any direct NGB role on autophagic flux activated by energetic stress, NGB upregulation appears functional in delaying stress-related cell death allowing an appropriate cell response and adaptation to the changing extracellular conditions.

## Introduction

During their life, cells may encounter unfavorable environmental conditions, which beyond a certain threshold became “stressors” activating the so-called stress response pathway, which, in turn, attempt to reduce cell damage and to maintain or re-establish cell homeostasis, or eventually eliminate damaged cells [1,2]. Stressor injury, like nutrient deprivation, hypoxia and oxidative stress, frequently occurs in living cells under either physiological or pathological states such as fasting, ischemia or solid tumor development [3].

In particular, cells triggered diverse strategies to cope with the fluctuation of nutrient availability including mobilization of stored (macro) molecules, recycling of cell components, and an overall reduction of functions [3]. Autophagy (macro-autophagy), an intracellular degradation pathway that occurs at basal levels in all cells during nutrient rich conditions, is one of the key cellular response upregulated in response to the nutrient withdrawal [4,5]. This process provides the cell with nutrients and energy by degrading cell components, by reducing the nutrient requirement, and decline of general functions; thus, autophagy allows cells to adapt themselves and function properly and coherently within the new environment. The failure of these strategies result in cells inability to respond properly and efficiently to stresses driving them to the apoptotic or necrotic death [3]. Pathological conditions, like solid cancer growth, conversely, are mainly linked to cells full adaption to the critical condition and escaping from the extracellular controls [6,7].

Neuroglobin (NGB) is an intracellular heme-globin. Several findings have supported a neuroprotective role of overexpressed NGB against hypoxic/ischemic and oxidative stress-related insults in both *in vitro* and *in vivo* experiments [8–14]. NGB operates as a mediator of stress sensing and cellular response coupling, in neuron-derived cells [10,15–17]. This role implies both the protein activation and/or its upregulation and the consequent triggering of adaptive cells response [10]. More recently, independent studies indicate that NGB protein level is differently modulated by oxidative stress and hypoxia in diverse extra nervous cancer cell lines and tissues [18,19]. In addition, we recently found NGB as a compensatory protein in the 17β-Estradiol (E2) activated pathway devoted to cell survival in both neuroblastoma (SK-N-BE) and primary neuron cells [8,20,21]as well as in extra nervous cancer cells [22–24]. Remarkably, as for neuron-derived cells, we demonstrated that NGB is a stress-inducible protein in breast cancer lines being upregulated in response to the oxidative stress, although low levels of O_2_ are unable to impact on the NGB expression [23]. Altogether, these results suggest that NGB exerts a pivotal role in sensing extracellular stimuli/stresses and in transducing information within the cells to mount an appropriate cellular response in both nervous and non-nervous cells. However, if NGB could play any role in the cell response to low nutrient availability, particularly regarding on the regulation of autophagic flux, is still unknown.

Here, the effect of nutrient deprivation condition on NGB expression and its impact on the downstream activated cellular response mechanisms, have been evaluated in neuroblastoma cells (SK-N-BE), breast cancer cells (MCF-7) and human embryonic kidney cells (HEK-293), cellular models sensitive to E2, which will be used as positive control on NGB levels and functions.

## Material and methods

### Reagents

E2, Pen-Strep solution, RPMI-1640 media without phenol red, Dulbecco’s modified Eagle medium (DMEM) without phenol red, Earle’s Balanced Salt Solution (EBSS), charcoal-stripped fetal calf serum, protease inhibitor cocktail, bovine serum albumin fraction V (BSA), Bafilomycin A1, anti-Tubulin and anti-LC3 antibodies and G418 (Geneticin) selection antibiotic were purchased from Sigma-Aldrich (St. Louis, MO, USA). Bradford protein assay was obtained from Bio-Rad Laboratories (Hercules, CA, USA). Anti-NGB, anti-Bcl2 and anti-p62 antibodies were obtained from Santa Cruz Biotechnology (Santa Cruz, CA, USA). The chemiluminescence reagent for Western blot super power ECL was obtained from Bio-Rad (Milan, Italy). All the other products were from Sigma-Aldrich. Analytical or reagent grade products were used without further purification.

### Cell culture

SK-N-BE and MCF-7 cell lines (ATCC, LGC Standards S.r.l., Milan, Italy) were used at passage 4–8 and were grown in air containing 5% CO2 in phenol red free, RPMI-1640 or DMEM medium, respectively, containing 10% (v/v) charcoal-stripped fetal bovine serum, L-glutamine (2.0 mM), Pen-Strep solution (penicillin 100 U/ml and streptomycin (100 mg/ml) as previously described [8,24] (Control Medium). Nutrient deprivation condition was obtained by culturing cells in amino acid and serum free, Earle’s Balanced Salt Solution (EBSS, Sigma Aldrich) containing 1 g/L of D-glucose for the indicated times. Stable ERα-transfected HEK-293 (ERα-HEK-293) cell lines were routinely grown in media containing G418 50 mM [25]. Cell line authentication were periodically performed by amplification of multiple STR loci by BMR Genomics srl (Padua, Italy). Cells were simultaneously treated with the vehicle used to dissolve all drugs (ethanol/PBS 1:10, v/v), and/or E2 (1 or 10 nM), and/or Bafalomycin-A1 (Baf-A1, 100 nM). When indicated, E2 or Baf-A1 pretreatment were performed adding the compounds 1 h before. For nutrient deprivation, MCF-7, SK-N-BE or ERα-HEK-293, were cultured as above reported, washed 3 times with PBS then cultured in EBSS for the indicated time points.

### Flag-NGB plasmid and cell transfection

The pcDNA-flag-NGB (Flag-NGB) was obtained by subcloning the NGB ORF from the NGBN1-pEGFP plasmid40 into the pcDNA-flag 3.1C. HEK-293 cells were grown to ~70% confluence and then transfected with pcDNA-flag-NGB plasmid using lipofectamine reagent (Invitrogen, Carlsbad, CA, USA) according to the manufacturer’s instructions. Four hours after transfection, the medium was changed and 24 h after the cells were treated as previously described.

### Western blot assay

Protein extraction and Western blot assay were performed as previously reported [21]. Briefly, after treatment, cells were lysed and solubilized in the YY buffer (50 mM HEPES at pH 7.5, 10% glycerol, 150 mMNaCl, 1% Triton X-100, 1 mM EDTA, 1 mM EGTA) containing 0.70% (w/v) SDS. Total proteins were quantified using the Bradford Protein Assay. Solubilized proteins (20 μg) were resolved by 10% or 15% SDS-PAGE at 100 V for 1 h at 24.0°C and then transferred to nitrocellulose with the Trans-Blot Turbo Transfer System (Bio-Rad, Hercules, CA) for 10 or 7 min, respectively. The nitrocellulose was treated with filtered 5% (w/v) BSA in 138.0 mM NaCl, 25.0 mM Tris, pH 8.0, at 24.0°C for 1 h and then probed overnight at 4.0°C with either anti-NGB (final dilution 1:1000), anti-LC3 (final dilution 1:1000), anti-p62 (final dilution 1:1000), anti Bcl-2 (final dilution 1:1000) and anti -Tubulin (final dilution 1:1000) antibodies. The antibody reaction was visualized with the chemiluminescence Western blotting detection reagent (Amersham Biosciences, Little Chalfont, UK). The densitometric analyses were performed by ImageJ software for Microsoft Windows (National Institutes of Health, Bethesda, MD, USA).

### Cell viability

ERα-HEK-293 cell lines transfected or not with Flag-NGB were grown to 70% confluence in 6-well plates and cultured with EBSS for the indicated time point. After treatment, cells were harvested with trypsin, and counted with Beckman Coulter Model ZM electronic particle (Palo Alto, Calif., USA).

### Statistical analysis

The statistical analysis was performed by Student’s t-test with the INSTAT software system for Windows. In all cases, p< 0.05 was considered significant.

## Results

### Effect of nutrient deprivation on NGB levels

**Fig 1:**
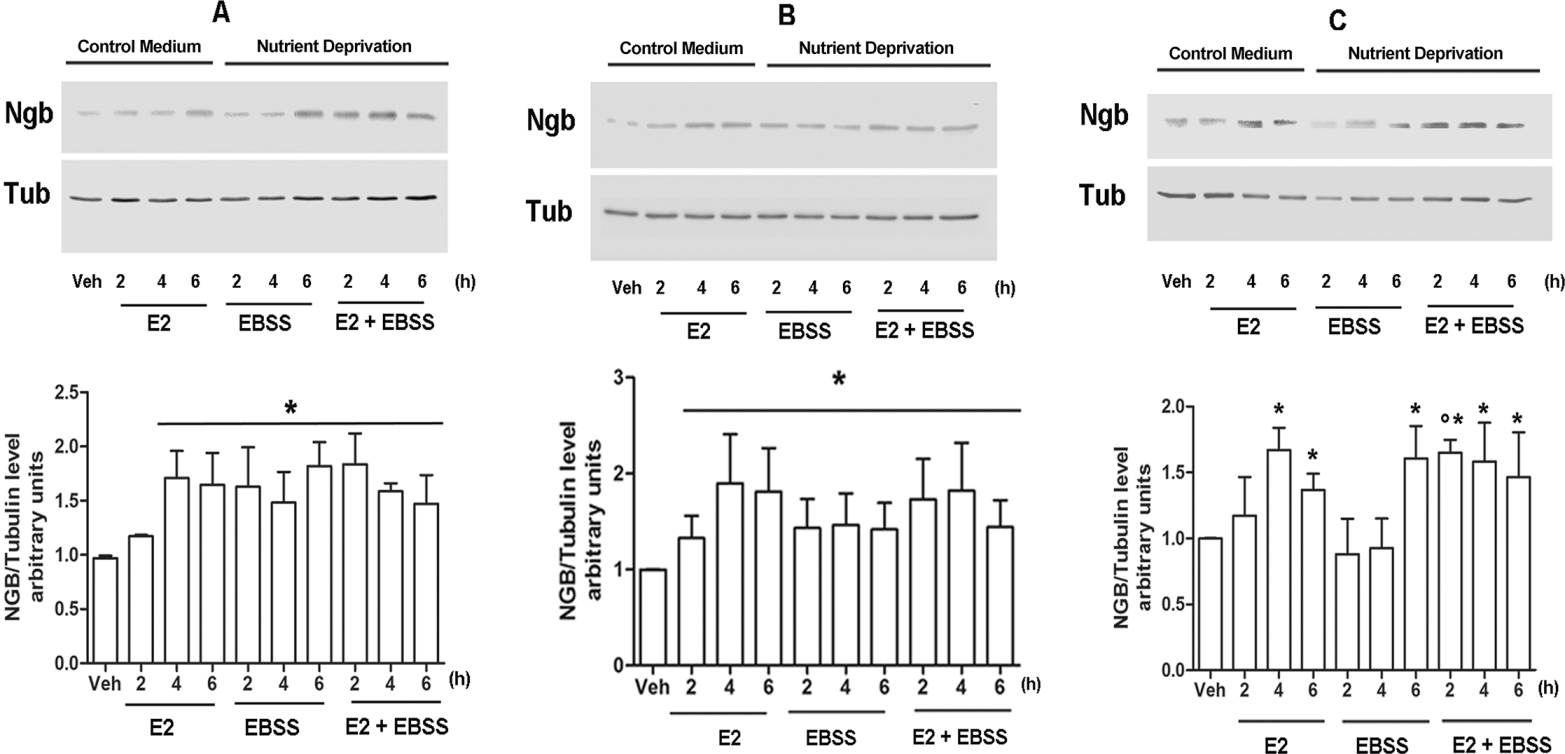
Nutrient deprivation impact on NGB expression levels. NGB protein expression in MCF-7 (A), SK-N-BE (B) and ERα-HEK-293 (C) cells cultured in control medium or in EBSS medium in presence or absence of E2 treatment (10 nM in MCF-7 and HEK-293 and 1 nM in SK-N-BE; 1 h pretreatment) or with E2 alone for 2, 4 or 6 h. The amount of protein was normalized to tubulin levels. Top panels are typical western blot of three independent experiments. Bottom panels represent the results of the densitometric analysis. Data are means ± SD of three different experiments. P<0.05 was determined with Student t-test vs. Veh (*) condition and vs E2 2h (°).

The effects of nutrient deprivation on the expression of NGB levels has been evaluated in human neuroblastoma cells (SK-N-BE) [8], breast cancer cells (MCF-7) [24] and the ER devoid HEK-293 [26] stable transfected with ERα plasmid (ERα-HEK-293). Cells were cultured in control medium or EBSS for 2, 4 and 6 h (Fig 1). E2 treatment (10 nM in MCF7 and ERα -HEK-293; 1 nM in SK-N-BE cells) has been used as positive control due to its well-known ability to upregulate NGB levels in these cell lines [8,22–24]. EBSS culturing increases NGB expression in all of the cell models considered (Fig 1A, 1B and 1C). Indeed, nutrient deprivation rapidly increases NGB level in SK-N-BE and MCF-7 cells (2h after the changing condition) while, in ERα-HEK-293 cells EBSS treatment enhances NGB expression just 6h after cell exposure, supporting a cell-based difference on stress response. In order to assess possible synergistic effects between E2 treatment and nutrient deprivation condition, cells were pre-treated with E2 (10 nM in MCF7 and ERα-HEK-293; 1 nM in SK-N-BE cells) 1h before the incubation with EBSS. In ERα-HEK-293, E2 shorten the time necessary to increase NGB levels (Fig 1C). However, E2 pre-treatment does not enhance EBSS-induced NGB upregulation in any of the considered cell lines (Fig 1).

### Nutrient deprivation and E2 effects on autophagic flux

**Fig 2:**
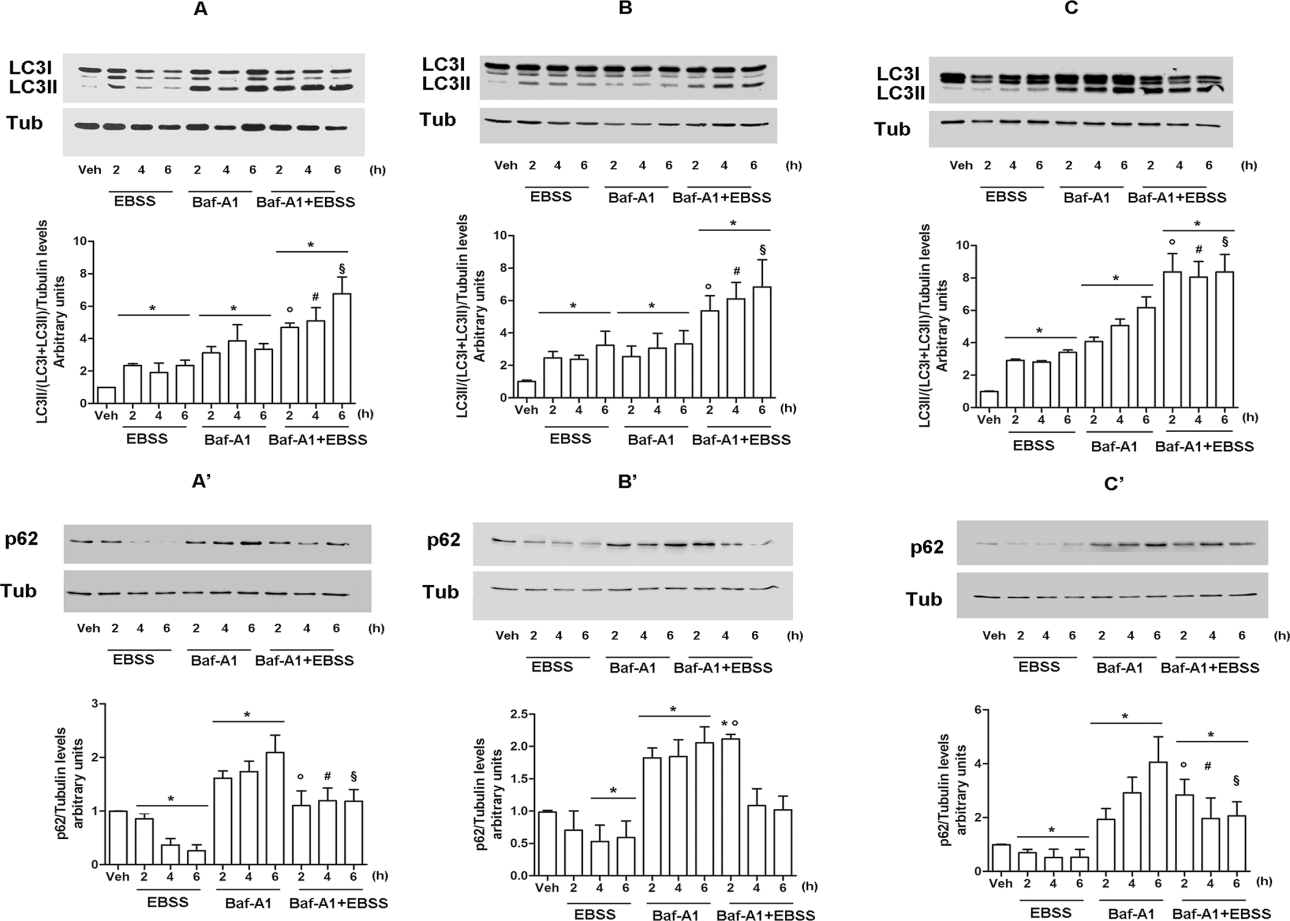
Nutrient deprivation induces autophagy flux. Western blot analysis of LC3 (A,B,C) and p62 (A’,B’,C’) expression in MCF-7 (A,A’), SK-N-BE (B, B’) and ERα- HEK-293 (C, C’) maintained in culture with control medium or with EBSS in presence or absence of Baf-A1 (100 nM, 1 h pretreatment) or with Baf-A1 (100 nM) alone at indicated time points. For LC3 quantitation, the formula LC3II/(LC3I + LC3II) has been applied. The loading control was done by evaluating tubulin expression in the same filter. Top panels are representative western blot of three independent experiments. Bottom panels are the relative results of densitometric analysis. Data are means ± SD of three different experiments. P<0.05 was determined with Student t-test vs Veh (*), EBSS 2h (°), EBSS 4h (#) and EBSS 6h (§) conditions.

Due to the strict relation between nutrient deprivation and autophagy [4,5], we next evaluated the activation of such cellular event in our cellular models at different time of nutrient deprivation exposure. Autophagy induction was evaluated by following the level of microtubule associated protein light chain 3 (LC3I) protein and its lipidated form (LC3II), which is the marker of the autophagosome number being accumulated on the autophagosome membrane where it remains until a complete degradation [27,28]. In all considered cell models, the amount of LC3II protein increases already 2 h after the exposure to nutrient deprivation suggesting a rapid cell response to the lack of nutrient availability. However, the accumulation of LC3II protein and, consequently, of autophagosomes, could be related to an effective increase of autophagy process and/or to a defect in the autophagolysosome formation associated with autophagy inhibition [27,28]. To solve this ambiguity, we analyzed the expression levels of p62 protein, also known as sequestrosome (SQSTM1), an autophagy cargo molecule that drive selected soluble molecules to the auto-phagolysosome for their degradation. Thus, p62 is considered as both an autophagy substrate and a marker of the autophagic flux being degraded when autophagy flux is allowed and accumulated when autophagy is impaired [27]. Fig 2 shows that the nutrient deprivation significantly leads to a rapid degradation of p62 protein parallel with the accumulation of LC3II protein sustaining an effective induction of autophagy flux induced by the EBSS culturing medium in selected cell lines. The cell treatment with the well-known autophagy inhibitor Baf-A1 (100 nM) [27] alone or 1h before the EBSS culturing resulted in a further LC3II accumulation (Fig 2). This additive effect indicates that nutrient deprivation-induced autophagosomes accumulation depends on the activation of autophagic flux not to its block.

**Fig 3:**
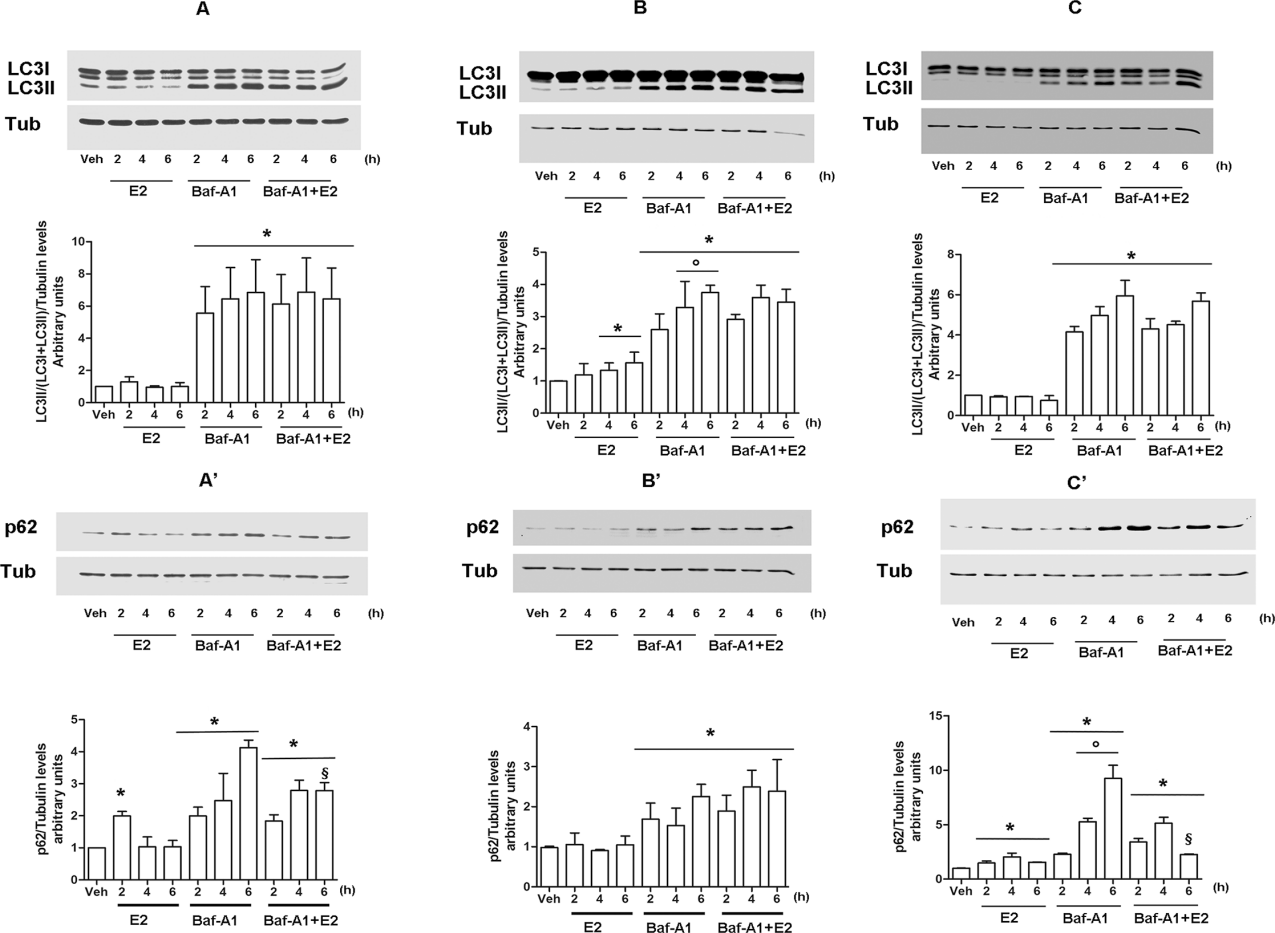
The effect of E2 on autophagy process. MCF-7 (A, A’), SK-N-BE (B, B’) and ERα- HEK-293 (C, C’) were treated with E2 (10nM in MCF-7 and ERα-HEK-293 and 1 nM in SK-N-BE) in presence or absence of Baf-A1 (100 nM; 1 h before) pretreatment, or with Baf-A1 (100 nM) alone at indicated time points. LC3 (A, B, and C) and p62 (A’, B’, C’) protein expression were analyzed via western blot. The amount of protein was normalized on tubulin levels. For LC3 quantitation, the formula LC3II/(LC3I + LC3II) has been applied. Top panels are representative western blots of three independent experiments. Bottom panels show results of densitometric analysis. Data are means ± SD of three different experiments. P<0.05 was determined with Student t-test vs Veh (*), E2 treated samples (°) and Baf-A1 6h (§) sample.

In order to evaluate if the other NGB inducer (i.e., E2) (see Fig 1) modulates the autophagic flux, E2 effects were assessed. As shown in Fig 3, E2 (10 nM) stimulation leads to the accumulation of p62 protein 2h after the hormone stimulation in both MCF-7 and in ERα-HEK-293 cells, without any significant effect on the expression of LC3II (Fig 3A, 3A’, 3C and 3C’). Such effect appears to be rapid and transient in MCF-7 where it vanishes 4h after E2 stimulation (Fig 3A’), whereas it persists until 6h in ERα-HEK-293 (Fig 3C’), probably due to the ectopic expression of ERα in these latter cells. On the other hand, in neuroblastoma SK-N-BE cells, E2 (1nM) treatment enhances the amount of LC3II protein since 4h after the stimulation (Fig 3B) without a parallel reduction of p62 protein (Fig 3B’), indicating that E2 leads to the accumulation of autophagosome without any completion of the autophagic flux. Altogether, obtained data indicate that E2 could affect autophagy flux exerting an inhibitory role on it. Notably, in all cells considered the Baf-A1 (100 nM) pre-treatment 1 h before E2 stimulation does not result in a further increase of LC3II and/or p62 protein levels respect to what observed with the Baf-A1 treatment alone (Fig 3). The absence of any additive effects stimulating cells with both E2 and Baf-A1 suggests a possible common mechanism shared by the hormone and the autophagy inhibitor in the last stage of autophagy flux.

### Involvement of NGB overexpression on the autophagic flux and cell viability during nutrient deprivation

**Fig 4:**
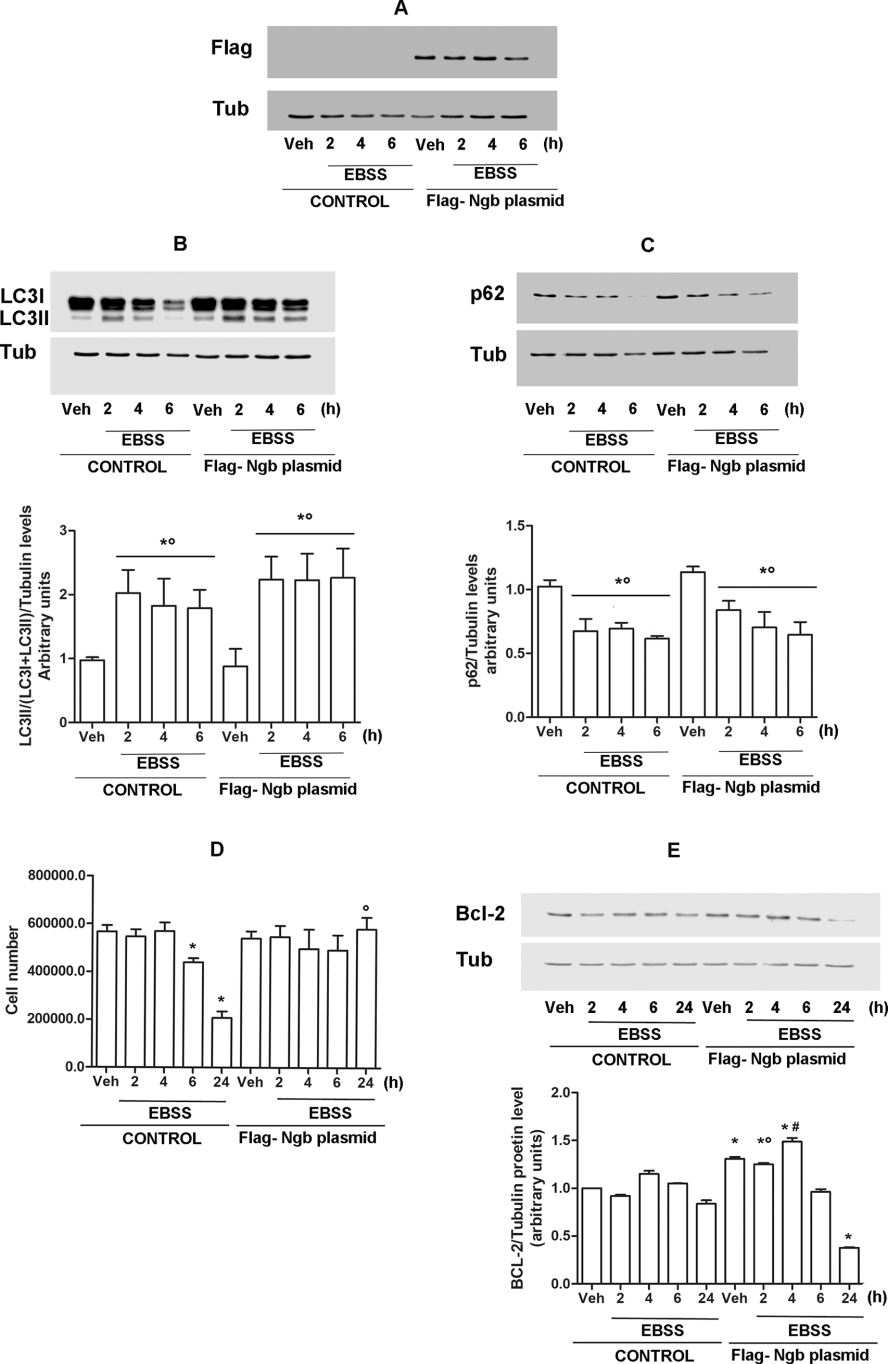
Impact of NGB overexpression on cell response to nutrient deprivation. ERα-HEK-293 cells were transiently transfected with Flag-NGB expressing plasmid. A) Western blot analysis of Flag expression in not transfected (CONTROL) and NGB-plasmid transfected (Flag-NGB plasmid) cells. Analysis of LC3 (B) and p62 (C) protein expression in not transfected and transfected cells cultured in control medium (10% FBS) or nutrient deprived EBSS solution for 2, 4 and 6h. The amount of protein was normalized on tubulin levels. For LC3 quantitation, the formula LC3II/(LC3I + LC3II) has been applied. Top panels are representative western blot of three independent experiments. Bottom panels are the relative results of densitometric analysis. Data are means ± SD of three different experiments. P<0.05 was determined with Student t-test vs CONTROL Veh (*) and Flag-NGB plasmid Veh (°). D) Evaluation of cell number during nutrient deprivation stress. Both not transfected (CONTROL) and NGB-plasmid transfected cells (Flag-NGB plasmid) were grown in control medium with 10% of FBS or in EBSS solution for 2, 4, 6 and 24h and counted at each time points. Data are means ± SD of four different experiments. P<0.05 was determined with Student t-test vs CONTROL Veh (*) and vs CONTROL EBSS 24h (°). E) Western blot analysis of Bcl-2 protein expression in not-transfected (CONTROL) and NGB-plasmid transfected cells (Flag-NGB plasmid) cultured in control medium (10% FBS) or EBSS at indicated time points. Top panel is representative western blot of three independent experiments. Bottom panel is the relative result of densitometric analysis. Data are means ± SD of three different experiments. P<0.05 was determined with Student t-test vs CONTROL Veh (*), CONTROL EBSS 2h (°) and CONTROL EBSS 4h (#).

Data reported in Figs 1 and 2 indicate that the EBSS-dependent up-regulation of NGB is not parallel with the induction of autophagy flux in ERα-HEK-293 cells where NGB increases after 6h, while autophagy is activated already 2h after. Such data lead to hypothesize that NGB induction is not directly linked to the autophagic flux activation. Therefore, in order to test the possible role of high levels of NGB in the autophagic process, ERα-HEK-293 cells were transiently transfected with pcDNA-Flag-NGB plasmid (Fig 4A). Both control and Flag-NGB overexpressing cells were exposed to nutrient deprivation condition for 2, 4 or 6h. Notably, the levels of both autophagy markers (i.e., LC3II and p62) do not show any significant difference between not–transfected and Flag-NGB transfected cells (Fig 4B and 4C). Furthermore, even the very high NGB levels reached by its ectopic expression 6h after nutrient deprivation does not change the level of the autophagy flux with the respect to the not-transfected cells supporting that a possible direct effect of NGB levels on autophagy activation, could be ruled out. Prolonged/chronic exposure to nutrient deprivation stress could lead to apoptotic or necrotic cell death [3]. This evidence prompted us to verify if Flag-NGB overexpression could affect cell viability during short and prolonged exposure to nutrient deprivation condition. Fig 4D shows that EBBS culturing significantly reduced cell number 6h after the treatment, which is parallel with the increase of NGB levels in this cell line (See Fig 1C). Such decline in cell number is further enhanced 24 h after exposure to nutrient deprivation condition. Remarkably, in Flag-NGB cells, EBSS-dependent decrease in cell number after 6 and 24h exposure is completely abolished (Fig 4D). Evidence have indicated that death induced by nutrient deprivation is commonly mediated by mitochondrial apoptotic pathway that mainly involves several members of Bcl-2 family, including the anti-apoptotic protein Bcl-2 [3]. This led us to evaluate the effect of overexpressed NGB on Bcl-2 expression. As reported in Fig 4E, in Flag-NGB ERα-HEK-293 cells the expression of Bcl-2 results significantly increased, with respect to the not-transfected counterpart, in both vehicle condition and until 4h after the exposure to nutrient deprivation. On the other hand, the Bcl-2 protein levels in Flag-NGB cells does not result significantly different from what observed in not-transfected cells after 6h nutrient deprivation exposure, or even reduced after 24h of low nutrient availability. Overall, these results show that ectopic expression of NGB increases cell survival during a prolonged exposure to nutrient deprivation, and it is linked to a parallel-enhanced expression of the anti-apoptotic protein Bcl-2.

## Discussion

NGB is a monomeric intracellular heme-globin, which attracted research interest in the last almost two decades because of its wide distribution in the brain and, in particular, of its well-known pro-survival effects against several type of extracellular insults when overexpressed [8–14]. In neuron derived cells and in extra nervous cancers, NGB expression is closely related and/or induced by stress conditions themselves like as hypoxia [29,30], oxidative stress (H_2_O_2_) [21,23,24], oxygen and glucose deprivation [31], and lipopolysaccharide treatment [20]. In addition, NGB ability to change its structure, reactivity, and function in response to intracellular redox state changes has further reinforced the idea that NGB, as a stress responsive-sensor, transfers the stress condition to the signal transduction pathways important for cell response to stress [17]. This evidence prompted us to evaluate the possible NGB modulation and function in the cell response to low nutrient availability. Indeed, although many research studies were aimed at evaluating the effect in neurons of ischemic injury and or oxygen and glucose deprivation on NGB expression [31–33], the role of nutrient stress on NGB protein levels is still unknown. Results reported here clearly indicate that culturing cells in starved condition positively modulates NGB protein levels in both neuroblastoma and breast cancer cell lines. In addition, such effects appear to share the same intracellular pathway activated by one of the main NGB inducer, E2, which does not exert any synergistic effects when given before the nutrient deprivation condition. Consistent with this, we recently prove that, at least in breast cancer cell line, both E2 and oxidative stress inducing compound Lead Acetate, led to the NGB expression through the activation of AKT pathway [34] (Fiocchetti et al. personal communication). It is possible that similar common mechanisms could be activated by nutrient deprivation to modulate NGB levels.

Nutrient withdrawal represents one of the main challenge that fast-growing cancer cells could encounter during cancer development [4]. The ability of cancer cells to adapt to stresses and to escape from cell death is fundamental for tumor growth and survival [4].

Autophagy is the key cellular responses that is promptly activated in response to nutrient stress [1,3–5]. It represents an homoestatic process based on the production of double membrane vesicle, autophagosomes, that expand to engulf citoplasmic components to degrade and use them as structure but also as an energy reserve [3,35,36]. Basal autophagy maintains protein and organelle quality control [36] and its rate is further enhanced during stressing condition to clear damaged organelles and recycle nutrients [3,35,36].

Accordingly, with literature, nutrient deprivation treatment leads to a rapid increase in the accumulation of the autophagosome marker LC3II with a parallel degradation of the p62 autophagy substrate in both neuroblastoma and breast cancer cells. Consistent with an effective increase in autophagy flux, by treating cells with autophagy inhibitor Baf-A1, a further increase in auto-phagosome accumulation during nutrient withdrawal occurs. On the contrary, E2 treatment blocks the continuation of autophagic flux in the cell model used. Despite of controversial evidence about the role of E2 on autophagy, showing the hormone ability to both induce [37,38] or block [39–41] autophagic flux, our data sustain the role of E2 as an anabolic hormone that, like as it occurs for insulin treatment [42], is expected to function suppressing the conserved catabolic process of self‑digestion. Moreover, the absence of any synergistic effects between E2 and Baf-A1 suggests that E2 and Baf-A1 share the same mechanisms in preventing the autophagic flux.

Differences between E2 and EBSS effects on autophagy lead to the paradoxical circumstance for which two different inducers of NGB overexpression oppositely affect the same intracellular mechanism. However, a possible direct involvement of up-regulated NGB in the general autophagy process could be kept out. Indeed, nutrient deprivation shows a different timing in the activation of autophagy process and the up-regulation of NGB protein levels, which sustains the lack of any direct relationship between such events. This is further confirmed by data demonstrating as the ectopic expression of NGB in ERα-HEK-293 cells does not change the autophagic flux markers (LC3II, p62) either in basal condition or after exposure to nutrient deprivation at those time points when the cellular response to nutrient withdrawal does not affect the NGB protein levels. Altogether, such evidence is consistent with other reported findings, which show that NGB overexpression does not exert any significant effect on the mRNA levels of upstream autophagy regulators Atg5, Atg7 and Beclin-1 [43].

Autophagy has been considered for long time a crucial process able to promote cancer survival under metabolic and genotoxic stresses which allows cancer resistance to treatment [4,35]. However, a mutually opposed survival and death-promoting role for autophagy has been suggested and mechanisms regulating such functions, in particular in cancer cells, are still far from resolution [4,35,36]. Remarkably, the E2 inhibitory effects on autophagy (present data) and its well-known anti-apoptotic functions in neuroblastoma [8] and breast cancer [24] cells, sustain the complexity of interrelationship between autophagy rate and cell death regulation.

As a whole, the lacking of any involvement of NGB overexpression in the autophagy activation does not rule out the possible role of stress upregulated NGB in the pro-survival mechanism activated by cells in response to extracellular nutrient deprivation. Indeed, an appropriate cell response to nutrient shortage it is not limited to the autophagy process, being generally pointed to attempt cell survival waiting for “better times” [3]. Accordingly, here we found that NGB overexpression preserves cell viability after prolonged exposure to nutrient withdrawal further sustaining the pro-survival role of high levels of NGB during different type of insults [8–10,24,44,45]. Nutrient deprivation-induced cell death mainly occurs through the activation of intrinsic or mitochondrial apoptotic pathway [3]. Such process relies on the balance between pro-apoptotic (i.e. Bim, Bax, Bid) and anti-apoptotic (i.e. Bcl-2, Bcl-xL, Bcl-x) [46] members of Bcl-2 protein family. As elsewhere reported [47], glucose withdrawal-induced death is activated via the mitochondrial translocation of Bax and, in MCF-7 cells, it can be inhibited by Bcl-2 overexpression [47]. Such evidence confirms that the increased expression of Bcl-2 protein is tightly linked to pro-survival and anti-apoptotic events. Furthermore, NGB expression is related to a decreased or increased expression of pro-apoptotic or anti-apoptotic Bcl-2 members, respectively [21,48–51]. In addition, E2-dependent up-regulation of NGB results pivotal in the hormone induced overexpression of Bcl-2 protein in breast cancer cell line [24]. Present reported findings show an increased level of Bcl-2 protein parallel with NGB overexpression. Therefore, although NGB may affect cell survival impinging on different pathways [17,21,51,52], the regulation of the Bcl-2 protein network may play a key role in the protective NGB function during energetic stress. In parallel, lowest Bcl-2 levels in NGB-overexpressed cells after a longer time of exposure to nutrient deprivation lead to hypothesize that the high levels of NGB could contrast cell death in a limited “time window” at the beginning of energetic stress-related insult. Indeed, stress-dependent induction of NGB could be functional to prevent accidental apoptosis during the exposure to low and shortened stress condition [52–55]. On the opposite, we recently proved as the E2-dependent up-regulation and mitochondrial re-localization of NGB is required to confer cell protection against high levels of oxidative stress [23,24].

A great amount of intracellular functions has been ascribed to overexpressed NGB, mainly linked to the well-known ability of the globin to exert pro-survival function [56–59]. Among the evidence put forward to define mechanisms underlying the protective function of NGB, several reports support a role of NGB in intracellular signaling impacting on metabolic, oxidative/hypoxia and survival/apoptotic pathways. NGB has been found upregulated by ischemia/hypoxia in cultured cell lines and primary mouse cortical neurons [10,33,29] and by oxidative stress [21] in neuroblastoma cell. In addition, NGB function as oxygen [15,60] and oxidative stress sensor [17] has been demonstrated. Overall, these data define a role of NGB as compensatory protein in the cell machinery activated in response to stress and as general stress adaptation marker of cancer cells susceptible to oxidative stress, oxygen and, as demonstrated here for the first time, even to nutrient willingness. Despite the lacking of any direct NGB role on autophagy flux activated by energetic stress, NGB upregulation appears functional in delaying stress-related cell death allowing an appropriate cell response and adaptation to the changing extracellular conditions. Therefore, NGB could represent a link between the cell ability to sense nutrient withdrawal, and the impairment of cell death during acute stress phase linked to the up-regulation of Bcl-2 anti-apoptotic protein.

Modulation of endogenous cellular defense mechanisms activated in response to stress represents an innovative approach in therapy in diseases causing chronic tissue damage, like as cancer [7]. Here reported observation add to our growing knowledge of the importance of NGB in mechanisms and structures involved in cellular stress response opening novel avenue in the development of therapeutic interventions.

## Abbreviations

Baf-A1: Bafilomycin A1
Bcl-2: B-cell lymphoma 2
BSA: bovine serum albumin fraction V
DMEM: Dulbecco’s modified Eagle medium
E2: 17β-estradiol
EBSS: Earle’s Balanced Salt Solution
ER: estrogen receptor
HIF-1α: hypoxia-inducible factor 1-alpha
H_2_O_2,_: hydrogen peroxide
LC3: Microtubule associated protein light chain 3
NGB: human neuroglobin
PI3K: phosphatidyl-inositol 3 kinase
SQSTM1: sequestrosome-1
